# Blood Transfusion Practices in Total Joint Arthroplasties in Jamaica

**DOI:** 10.4021/jocmr2009.12.1279

**Published:** 2009-12-28

**Authors:** RE Christopher Rose, Ayana Crichlow, Christine Walters, Andrew Ameerally, Georgiana Gordon-Strachan

**Affiliations:** a Division of Orthopaedics, Department of Surgery, Radiology, Anesthesia and Intensive Care, The University of the West Indies, Mona, Kingston 7, Jamaica; b Health Research Resource Centre, Office of the Dean, Faculty of Medical Sciences, The University of the West Indies, Mona, Kingston 7, Jamaica

## Abstract

**Background:**

Major blood loss usually occurs in both hip and knee arthroplasty, frequently leading to the need for blood transfusion. This study was performed to determine blood transfusion rates and analyze the factors which affected the need for blood transfusion in patients who underwent primary unilateral total knee and hip arthroplasties at the University Hospital of the West Indies, Jamaica.

**Methods:**

A prospective study of 118 patients who underwent unilateral total knee and total hip arthroplasties between January 2004 and July 2009 was undertaken. Data collected was analyzed using Microsoft Excel 2008, SPSS version 12 and Stata version 7.0.

**Results:**

Of the 118 patients, 90 (70%) were females. Mean ± standard deviation (SD) age was 65.2 ± 11.5 years (range 32 - 85 years). Osteoarthritis accounted for the majority (88%) of arthroplasties. Mean ± SD estimated blood loss for all arthroplasties was 1195.0 ± 855.6 ml (range 100 - 6000 ml). Mean ± SD duration of surgery for all joint arthroplasties was 226.1 ± 63.5 minutes (range 110 - 392 minutes). Mean ± SD preoperative hemoglobin was 12.09 g/dl (range 7.3 - 15.6 g/dl). Average body mass index was 28.9 kg/m^2^ (range 17.9 - 68.3 kg/m^2^). Seventy-five (64%) patients were transfused and of these, 44 patients received allogenic blood only; 20 patients received autologous blood only, and eleven patients received both allogenic and autologous blood.  The overall blood transfusion rate was 63%.

**Conclusion:**

In our study, the multivariate analysis showed a significant relationship (p = 0.000) only between postoperative transfusion and the estimated blood loss.

**Keywords:**

Blood transfusion practices; Total joint arthroplasties

## Introduction

Total knee and hip arthroplasties are usually associated with significant blood loss and many patients require perioperative transfusion [1,2]. The reported mean estimated blood loss is between 495 and 1,493ml per procedure [3-5]. Allogenic transfusion is associated with a number of problems, including the risk of transmission of infectious diseases such as Human Immunodeficiency Virus and hepatitis, as well as transfusion-related and allergic reactions [6-8]. In addition, some patients refuse a blood transfusion because of religious beliefs. In order to minimize the disadvantages of allogenic transfusions, alternatives such as preoperative haemodilution, anesthetic hypotension, intraoperative and postoperative blood recovery, preoperative donation of blood, and human recombinant erythropoietin, are currently being used [9-11].

The aim of this prospective study was to determine blood transfusion rates and analyze the factors which affected the need for blood transfusion in patients who underwent primary unilateral total knee and hip arthroplasties at the University Hospital of West Indies, Jamaica.

## Materials and Methods

Between January 2004 and July 2009, 148 consecutive patients who underwent total knee and total hip arthroplasties by three orthopaedic surgeons at the University Hospital of the West Indies in Jamaica were prospectively studied. Patients were excluded from the study if they were undergoing total joint arthroplasty for a hip fracture; were undergoing revision surgery; had a hematological disease; were being treated with warfarin for anticoagulation; had liver impairment; had had gastro-intestinal bleeding; were undergoing simultaneous joint arthroplasties or received a transfusion in the preoperative period. There were seven bilateral total joint arthroplasties; 19 revisions; two patients with liver impairment, and two patients with a history of gastro-intestinal bleeding. The study population consisted of 118 unilateral primary total joint arthroplasties. There were ten patients each of whom underwent two procedures during the study. Informed consent was obtained from all of the patients. Of the ten patients, five patients each had two primary total knee arthroplasties; three patients each had two primary total hip arthroplasties, and two patients each had a primary total hip and primary total knee arthroplasty. The range of time between the procedures was seven days to 69 months. The following information was recorded for each patient; diagnosis, procedure, age, gender, body mass index (BMI), duration of surgery, preoperative hemoglobin, estimated blood loss, type of anesthesia, number of predonated autologous units of blood, number of patients transfused and the number of units of blood discarded.

Data collected was analyzed using Microsoft Excel 2008, SPSS version 12 and Stata version 7.0. Descriptive statistics were generated as appropriate. Student t-tests were used to compare average values for continuous variables. Logistic regression models were also used to examine which variables were predictive of the risk for post-surgery transfusion.  Statistical significance for all tests was set at 5%.

## Results

Of the 118 patients, 90 (76%) were females. The mean ± standard deviation (SD) age was 65.2 ± 11.5 years (range 32 - 85 years). One hundred and eighteen total joint arthroplasties were performed of which 63 were total knees and 55 were total hips. The gender distribution of the joint arthroplasties was 51 (81%) females in the total knee arthroplasty group, and 39 (71%) females in the total hip arthroplasty group. The diagnosis of the patients who underwent total knee and hip arthroplasties were as follows: osteoarthritis accounted for 104 arthroplasties; rheumatoid arthritis for nine arthroplasties; systemic lupus erythematosis for three arthroplasties and avascular necrosis for seven arthroplasties. Five patients had more than one diagnosis. Average body mass index (or BMI) for the 104 patients with available information was 28.9 kilograms per meters squared (kg/m^2^), ranging from17.9 to 68.3 kg/m^2^. The mean ± SD duration of surgery for all joint arthroplasties was 226.1 ± 63.5 minutes (range 110 - 392 minutes). The mean ± SD duration of surgery for total knee arthroplasties was 225.9 ± 66.2 minutes (range 115 -392 minutes). The mean ± SD duration of surgery for total hip arthroplasties was 226.4 ± 60.9 minutes (range 110 - 360 minutes).

The mean ± SD estimated blood loss for all arthroplasties was 1195.0 ± 855.6 ml (range 100 - 6000 ml). The mean blood loss in total knee arthroplasties was 1,106.2 ± 645.1 ml (range 100 - 3,200 ml). The mean blood loss in total hip arthroplasties was 1,295.1 ± 1,040.8 ml (range 200 - 6000 ml). One patient who underwent a total hip arthroplasty had an estimated blood loss of 6 liters. The types of anesthesia utilized were 43 general anesthesia, 57 regional anesthesia, and 18 combined regional and general anesthesia.

**Figure 1:**
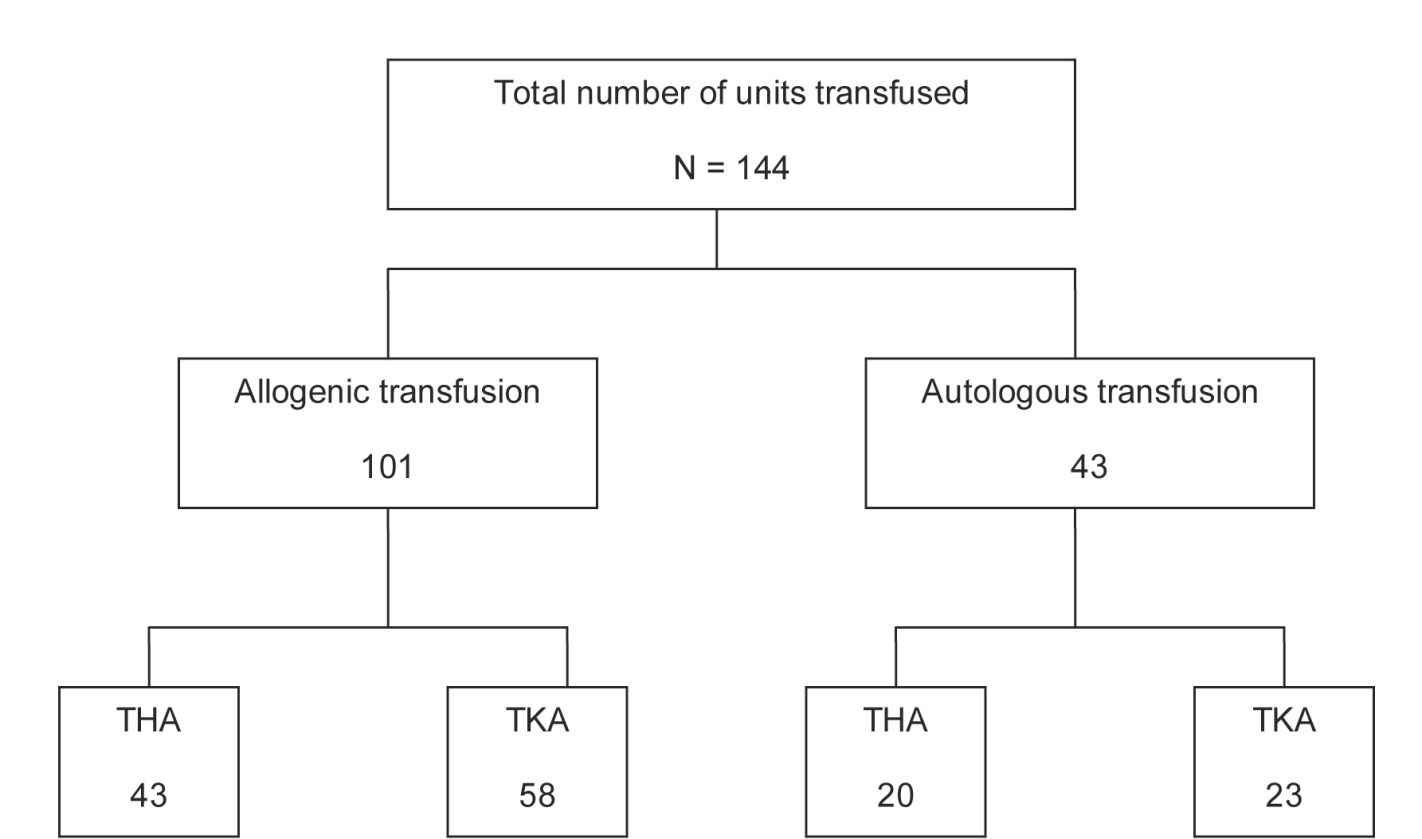
Blood transfusion requirements. THA (total hip arthroplasty), TKA (total knee arthroplasty).

The mean ± SD preoperative hemoglobin was 12.0 ± 1.4 g/dl (range 7.3 - 15.6 g/dl). Seventy-five (64%) patients were transfused and of these patients 44 patients received allogenic blood only; 20 patients were transfused autologous blood only and eleven patients received both allogenic and autologous blood. Thirty-six (30%) patients predonated a total of 46 units of blood. Of the 75 patients who were transfused, 33 (44%) donated autologous blood. A total of 144 units of blood were transfused with 101 units being allogenic transfusions. The number of units transfused was higher for the total knee arthroplasty group (81 units), than for the total hip arthroplasty group (63 units) (Fig. 1).

The overall blood transfusion rate was 63% (total knee arthroplasty 67%; total hip arthroplasty 60%).  Number of units transfused per procedure was approximately 2:1.

A total of 6 units of blood were unused (3 units of autologous blood and 3 units of allogenic blood). Table 1 shows the results of multivariable logistic regression to assess whether preoperative hemoglobin levels, age, gender, BMI, duration of surgery, estimated blood loss, and type of anesthesia were predictive of risk for post-surgery transfusion.

**Table 1:** Logistic Regression Analysis to Assess Factors Impacting the Risk of Post-surgery Transfusion

Variable	Odds Ratio	95% CI	P-value
Gender (M vs F)	0.544	0.153 - 1.936	0.347
Age	1.005	0.960 - 1.053	0.815
Pre-op Hb	1.289	0.865 - 1.921	0.212
BMI	1.046	0.932 - 1.174	0.447
Duration of surgery	1.005	0.995 - 1.015	0.354
Est. blood loss	1.002	1.001 - 1.003	0.000*
Type of anaesthesia			
Regional vs General	2.033	0.620 - 6.668	0.242
Combined vs General	1.026	0.220 - 4.785	0.974

N = 102 patients with non-missing values.

**Figure 2:**
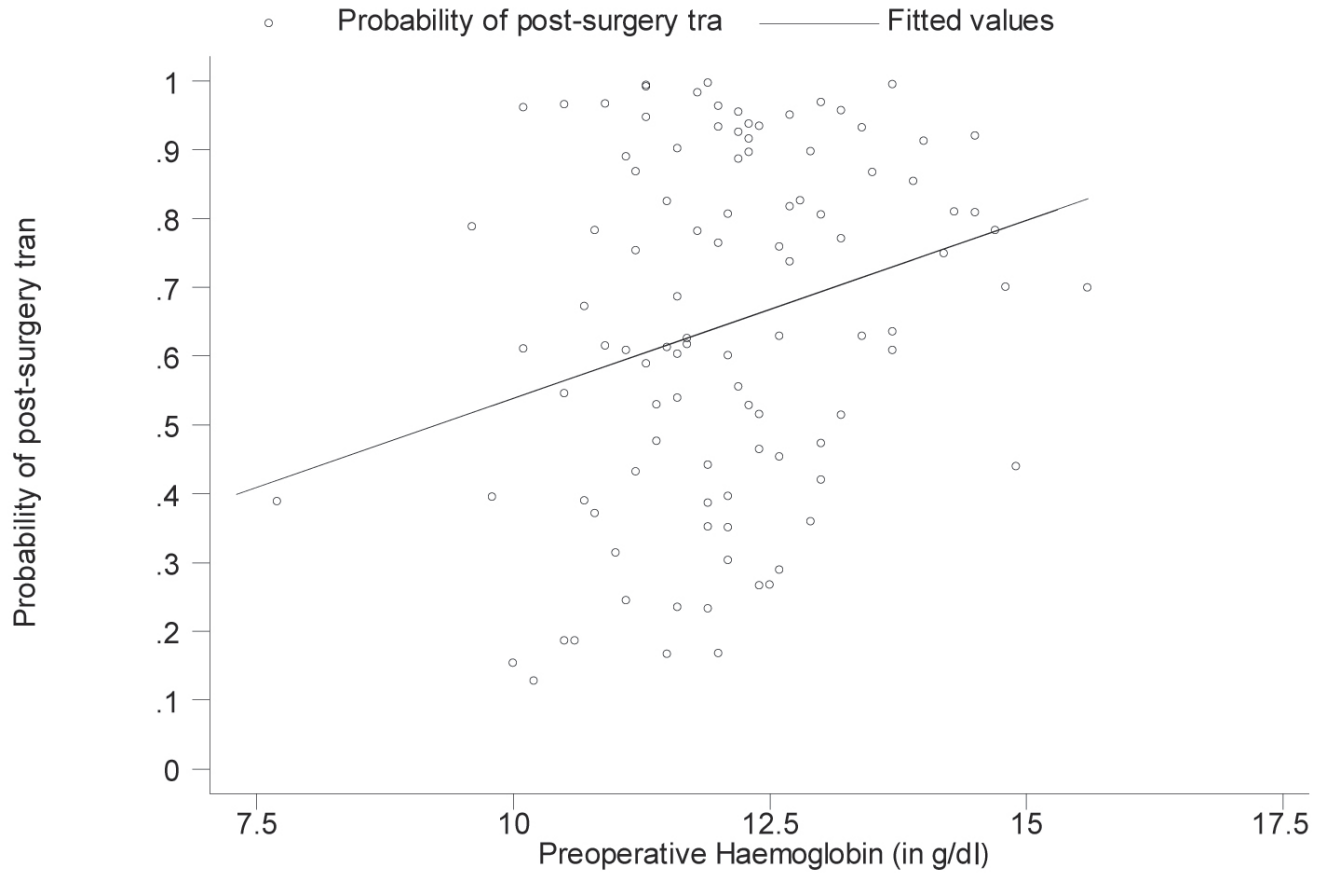
Graphing the predicted risk of post-surgery transfusion against preoperative hemoglobin values (adjusted for all the other variables listed in Table 1).

Predicted risk of post-surgery transfusion, generated based on the logistic regression model in Table 1, were plotted against preoperative hemoglobin levels (Fig. 2). A fitted linear regression line was super-imposed, showing a direct relationship between predicted risk of post-surgery transfusion and preoperative hemoglobin levels after adjusting for gender, age, BMI, duration of surgery, estimated blood loss, and type of anesthesia.

Thirty-six patients donated blood, and 33 of these patients were transfused with autologous blood post-surgery. Table 2 summarizes the multivariable logistic regression when the 36 patients who donated blood were excluded.

**Table 2:** Logistic Regression Analysis to Assess Factors Impacting the Risk of Post-surgery Transfusion

Variable	Odds Ratio	95% CI	P Value
Gender (M vs F)	0.277	0.032 - 2.393	0.243
Age	1.015	0.951 - 1.084	0.651
Pre-op Hb	0.777	0.413 - 1.463	0.435
BMI	1.107	0.954 - 1.286	0.181
Duration of surgery	1.007	0.993 - 1.022	0.329
Est. blood loss	1.002	1.001 - 1.004	0.002*
Type of anaesthesia			
Regional vs General	2.568	0.554 - 11.889	0.228
Combined vs General	1.474	0.042 - 5.285	0.544

N = 68 patients with non-missing values and who did not donate blood.

**Figure 3:**
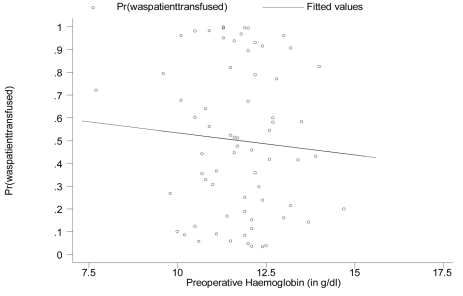
Graphing the predicted risk of post-surgery transfusion against preoperative hemoglobin values (adjusted for all the other variables listed above where patients who donated autologous blood are excluded).

As done previously, predicted risk of post-surgery transfusion, generated based on the logistic regression model in Table 2, were plotted against preoperative hemoglobin levels (Fig. 3). The fitted linear regression line showed an inverse relationship between predicted risk of post-surgery transfusion and preoperative hemoglobin levels after adjusting for gender, age, BMI, duration of surgery, estimated blood loss, and type of anesthesia.

Based on t-tests for the comparison of means, average preoperative hemoglobin values did not differ significantly between persons who were or were not transfused post-surgery. That finding was consistent within gender as well as when persons who donated autologous blood were excluded. Among males who did not donate autologous blood and then were later transfused, their mean preoperative hemoglobin was 12.0 g/dl, compared to 11.6 g/dl among their female counterparts (P-value = 0.575, not significant). Males who did not donate autologous blood and were never transfused had an average preoperative hemoglobin of 13.1 g/dl compared to 11.5 g/dl for their female counterparts (P < 0.001, significant) (Tables 3, 4)

**Table 3:** Analysis of Average Preoperative Hemoglobin Levels in all Patients

Was patient transfused?	Number of patients	Pre-operative Hemoglobin			
		Mean	Standard Deviation	95% CI	P-value
All Patients:					0.359
No	43	11.9	1.3	11.5 - 12.3	
Yes	75	12.1	1.4	11.8 - 12.4	
Females only					0.094
No	32	11.5	1.0	11.1 - 11.9	
Yes	58	11.9	1.3	11.6 - 12.3	
Males only					0.555
No	11	13.0	1.2	12.2 - 13.9		
Yes	17	12.7	1.5	11.9 - 13.5	

**Table 4:** Analysis of Average Preoperative Hemoglobin Levels in Persons who did not Donate Blood

Was patient transfused?	Number of patients	Pre-operative Hemoglobin			
		Mean	Standard Deviation	95% CI	P-value
All Patients:					0.462
No	40	11.9	1.3	11.5 - 12.3	
Yes	42	11.7	1.3	11.2 - 12.1	
Females only					0.730
No	31	11.5	1.0	11.1 - 11.9	
Yes	37	11.6	1.3	11.2 - 12.1	
Males only					0.154
No	9	13.1	1.4	12.1 - 14.2	
Yes	5	12.0	1.3	10.3 - 13.6	

## Discussion

A patients risk of requiring a transfusion during surgery and in the immediate postoperative period is an important element of effective blood management. Estimated perioperative blood loss and preoperative hemoglobin concentration are critical predictors of the need for blood transfusion. In our study, the multivariate analysis showed a significant relationship only between postoperative transfusion and the estimated blood loss (p = 0.000). Table 1 revealed that persons were 28.9% more likely to need post-surgery transfusion given a 1g/dl increase in preoperative hemoglobin levels. However, this value was non-significant (95% confidence interval (CI) for the odds ratio: 0.865 - 1.921, p = 0.212). From Table 2, although the odds ratio for preoperative hemoglobin levels remained non-significant (95% CI: 0.413 - 1.463; p = 0.435), there is a reversal in the direction of its impact on the risk for post-surgery transfusion. Persons were now 22.3% less likely to need post-surgery transfusion given a 1g/dl increase in preoperative hemoglobin levels, after adjusting for gender, age, BMI, surgery duration, estimated blood loss during surgery and type of anesthesia used. These results are not consistent with the findings in the literature. Several studies have established a relationship between the preoperative hemoglobin level and the need for postoperative blood transfusion [3-5, 12]. The abnormal finding in our study can possibly be explained by the absence of hemoglobin levels immediately preoperative in some of the patients who predonated blood. These patients, after predonation, possibly had low preoperative hemoglobin levels. Thirty-three of 36 patients who predonated blood were transfused their autologous blood post-surgery. The results would be skewed since the analysis was performed using predonation hemoglobin levels rather than immediate preoperative hemoglobin levels in some of the patients who predonated blood. The multivariable analysis with the exclusion of the 36 patients who donated blood revealed that the preoperative hemoglobin levels remained non-significant but there was a reversal in the direction of its impact on the need for post-surgery transfusion.

Salido et al [12] reported that patients with a preoperative hemoglobin level less that 13 g/dl had a four times greater risk of having a transfusion than did those with a hemoglobin level between 13 g/dl and 15 g/dl, and a 15.3 times greater risk than did those with a hemoglobin level of greater than 15 g/dl. Bierbaum et al [3] also reported that the rate of breakthrough transfusion was highest for their patients who had a baseline hemoglobin that was between 10 g/dl and 13 g/dl.

Salido et al [12] and Bierbaum et al [3] found that the prevalence of blood transfusion after hip surgery was higher than that after knee surgery, although this difference was not significant. In our study, the blood transfusion rate was higher in knee surgery (67%) than hip surgery (60%). The average blood transfusion rates for total knee and hip arthroplasties in studies by Bierbaum et al [3], Feagan et al [13], and Pierson et al [4] were 49%, 27% and 2.1% respectively. Our transfusion rate was 63%. There was no standard protocol for proceeding with a transfusion in our study. Instead, the decision for blood transfusion was based on the patients overall clinical status and co-morbidities.  Many authors advocate that postoperative transfusion trigger can be brought to 8 g/dl in a hemodynamically stable patient [5,14,15].

Bierbaum et al [3] confirmed in their study that allogenic blood was transfused despite extensive use of predonated autologous blood. Twenty percent (796) of the 3,920 patients who had a procedure on the hip and 13% (723) of the 5,552 who had a procedure on the knee had a transfusion of allogenic blood. The rates of transfusion of allogenic blood were highest for the patients who had a revision or a bilateral procedure and for those who had a baseline hemoglobin level of 13 g/dl or less. In our study, of the 75 patients who were transfused, 44 (59%) received allogenic blood. A total of 144 units of blood were transfused with 101 units being allogenic transfusions.

The use of preoperative autologous blood donation has increased substantially although it has been associated with such risks as preoperative anaemia, ischemic events, and complications severe enough to require hospitalization [16,17]. Furthermore, the magnitude and rate of patient response to compensatory erythropoiesis to replace donated red blood cells generally has been overestimated [18]. In one study [19], preoperative donation between 42 and seven days before surgery still resulted in an average decrease of 1g/dl for every unit of autologous blood donated, suggesting an absence of adequate compensatory erythropoiesis. Over collection of blood is also a problem associated with preoperative autologous donation. As much as 50% of autologous blood is unused in patients undergoing joint replacement [18]. Bierbaum et al [3] found that a high percentage of autologous blood was discarded, and many patients never received any of there predonated units. In our study, 36 (30%) of 118 patients predonated a total of 46 units of blood. Three units of autologous blood were unused. Two units were donated by one patient who had tested positive for HIV. One patient refused autologous transfusion.

A major weakness of this study is the small study population. The University Hospital of the West Indies is a low volume centre for total hip and knee arthroplasties. The high trauma burden on our operating lists, the limited operating time and the great consumption of blood by trauma patients are the main reasons for the small numbers in this paper. Our results have shown that there is no relationship between age, gender, BMI, duration of surgery, type of anesthesia and preoperative hemoglobin level and the need for postoperative blood transfusion. There was a significant relationship between the estimated blood loss and the need for postoperative blood transfusion.
